# The surprising role of stimulus modality in the dual-task introspective blind spot: a memory account

**DOI:** 10.1007/s00426-021-01545-y

**Published:** 2021-07-13

**Authors:** Donna Bryce, Daniel Bratzke

**Affiliations:** 1 Department of Psychology, University of Tübingen, Schleichstrasse 4, 72076 Tübingen, Germany; 2 Department of Psychology, University of Bremen, Hochschulring 18, 28359 Bremen, Germany

## Abstract

Being able to accumulate accurate information about one’s own performance is important in everyday contexts, and arguably particularly so in complex multitasking contexts. Thus, the observation of a glaring gap in participants’ introspection regarding their own reaction time costs in a concurrent dual-task context is deserving of closer examination. This so-called introspective blind spot has been explained by a ‘consciousness bottleneck’ which states that while attention is occupied by one task, participants cannot consciously perceive another stimulus presented in that time. In the current study, a series of introspective Psychological Refractory Period (PRP) experiments were conducted to identify the determinants of an introspective blind spot; to our surprise, in half of the experiments participants appeared to be aware of their dual-task costs. A single trial analysis highlighted the sensory modality of the two stimuli within the trial as an important predictor of introspective accuracy, along with temporal gaps in the trial. The current findings call into question the claim that attention is required for conscious awareness. We propose a memory-based account of introspective processes in this context, whereby introspective accuracy is determined by the memory systems involved in encoding and rehearsing memory traces. This model of the conditions required to build up accurate representations of our performance may have far-reaching consequences for monitoring and introspection across a range of tasks.

## Introduction

The study of how accurately people can introspect about their own performance in complex tasks is of the utmost importance. Flawed introspection about our abilities could have considerable real-life consequences, such as risk-taking when we misjudge our own limitations (e.g. in multitasking as in Finley et al., 2014). The field of metacognition occupies itself with this topic primarily because accurate introspection (or metacognitive monitoring) can contribute to improving performance (monitoring-based regulation, e.g. Koriat & Goldsmith, 1996; Koriat et al., 2006). The study of the content of introspection in multitasking also has the potential to inform us directly about the relationship between consciousness and attention, as it has been proposed that while attention is limited, experiences remain inaccessible to introspection (preconscious; Dehaene et al., 2006).

A particularly fruitful approach to understanding what determines the accuracy of introspection is to study situations in which there are glaring gaps in one’s introspection about their own performance. One such situation is during dual-task performance, in which participants appear to be unaware of the costs associated with processing two tasks concurrently (a so-called ‘introspective blind spot’, first reported by Corallo et al., 2008). Interestingly, people seem to have no such difficulty introspecting about their task-switching performance (Bratzke & Bryce, 2019), suggesting that the introspective blind spot does not occur in all types of multitasking. Here, in a series of experiments we investigate the conditions required to elicit the introspective blind spot in dual tasking, with the wider aim of understanding more about the composition of introspection in attentionally demanding tasks. To our surprise, the results highlighted the importance of a factor that has until now received little or no attention in this field - the modality of each stimulus present in the trial.

**Fig. 1:**
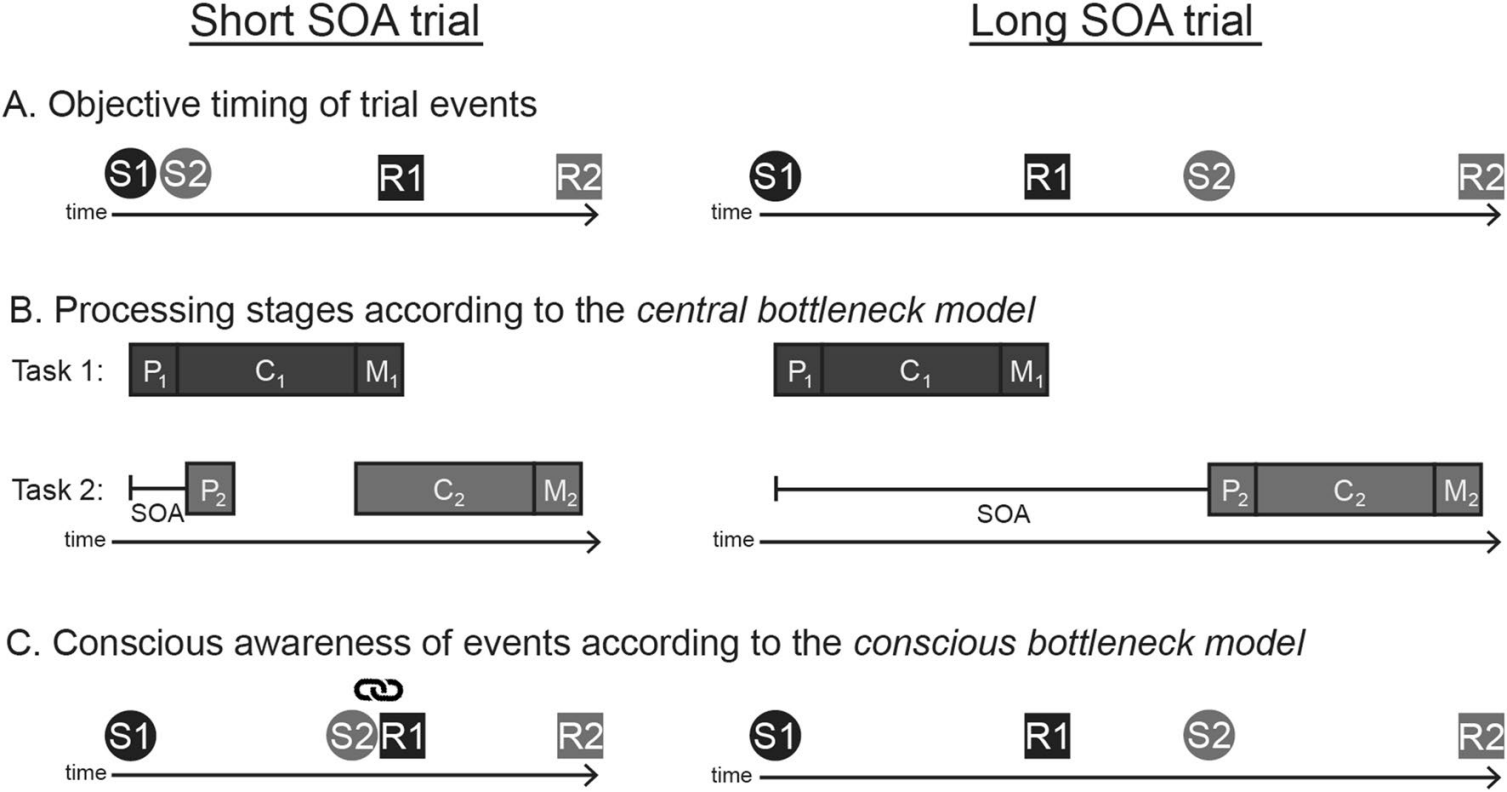
Illustration of the objective timing of events (**A**), the central bottleneck model (**B**) and conscious awareness according to the conscious bottleneck model (**C**) in a trial with a short stimulus onset asynchrony (SOA; left side) and a long SOA (right side). Here and in the text, S1 and S2 refer to the stimuli for Task 1 and Task 2, respectively, and R1 and R2 refer to the responses for Task 1 and Task 2, respectively. The central bottleneck model states that each task has three processing stages: perceptual (P), central (C) and motor (M), all of which can proceed in parallel except for the two central stages, which cannot occur simultaneously. Note that according to the central bottleneck model, RT2 is longer in short than long SOA trials because central processing of Task 2 (C_2_) must wait until central processing of Task 1 (C_1_) is completed. According to the conscious bottleneck model, S2 cannot reach conscious awareness until central processing of Task 1 is completed. Accordingly, in the short SOA trial S2 is linked to the end of Task 1 central processing (and, therefore, to R1). This is illustrated by the link symbol

Numerous introspective Psychological Refractory Period (PRP) experiments have observed an introspective blind spot in dual-task contexts (Bratzke & Bryce, 2016; Bratzke et al., 2014; Bryce & Bratzke, 2014, 2015, 2017; Corallo et al., 2008; Marti et al., 2010); that is, participants are unaware of the dual-task costs on their reaction times. In PRP experiments, participants must respond to two choice reaction time tasks, which can be presented close together in time (with a short stimulus onset asynchrony, SOA) or with a longer gap between them (a long SOA; see Fig. 1A). The typical behavioural finding is that response times to the second task (RT2) are much longer when the tasks are processed concurrently than sequentially (short vs. long SOA; the PRP effect), whereas response times to the first task (RT1) are unaffected by SOA (Pashler, 1994). The RT effects elicited in the PRP paradigm are well explained by the central bottleneck model (Pashler, 1994) which states that while perceptual and motor processing of two tasks can occur in parallel, central processing (e.g. response selection) is strictly serial (see Fig. 1B). In introspective PRP experiments, participants estimate their RTs to each task after each trial (referred to as introspective RTs, iRTs). The typical introspective finding is that neither iRT1 nor iRT2 is affected by SOA, and, therefore, participants appear to be unaware of the dual-task costs in this context (even though other RT effects can be reported, see, e.g. Bryce & Bratzke, 2014; Bratzke & Janczyk, 2021).

The original explanation for the unawareness of the PRP effect offered by Marti et al. (2010; here named the ‘conscious bottleneck model’) was that participants specifically misperceive the onset of the second stimulus (S2) in the short SOA condition, as it cannot be consciously perceived while Task 1 is being centrally processed. Thus, when the processing of the two tasks is concurrent at short SOAs, the central processing bottleneck that causes the PRP effect also places a structural limitation on conscious awareness. That is, the central bottleneck renders the second stimulus preconscious while attentional resources are occupied by Task 1 central processing (see Fig. 1C for an illustration of the misperception of S2 in short SOA trials according to the conscious bottleneck model). Thus, the unawareness of the PRP effect has been viewed as evidence that top–down attention is required for conscious perception, as stated in the taxonomy of conscious perception proposed by Dehaene et al. (2006).

The majority of the evidence supporting the idea that the second stimulus is misperceived at short SOAs was collected using visual analogue scales (VAS; participants give their estimates by clicking on a horizontal line labelled at each end with numerical values). That is, after each trial, Marti and colleagues collected separate estimates of various intervals in the preceding trial (i.e. RT1, RT2, SOA, delay between S2 and decision about S1) and then reconstructed the subjective phenomenology of the PRP trial. However, a more recent study cast doubt on the reliability of iRTs provided via VASs (Bryce & Bratzke, 2017). In this study, yoked trials were ‘replayed’ to participants and their only task was to separately estimate the intervals that represented RT1 and RT2. That is, real trials from other participants were replayed to new participants and they completed this task as a pure timing task with no PRP processing demands. Even when participants were not processing the PRP task, they could not report the intervals that represent RTs accurately using the VAS method. This raises questions about the suitability of reconstructing participants’ introspective representation of a trial using separate VAS estimates.

An alternative method, the timeline, which more accurately and in more detail depicts participants' introspective representation of a trial was developed by Bryce and Bratzke (2017). Participants recreated the trial by placing markers that represent each trial event on a timeline (and thus directly reported their subjective phenomenology of each PRP trial). Using this method, participants could faithfully recreate the PRP effect when there were no PRP processing demands, but could not when they first had to process the PRP trial. An advantage of the timeline method is that it allows us to examine introspective accuracy about the whole trial rather than focussing only on iRT2, meaning the data can be interrogated for evidence regarding the aforementioned conscious bottleneck model. While the existence of an introspective blind spot during dual-task processing was indeed still supported using the timeline method, there was no evidence that the conscious perception of the second stimulus was specifically delayed in short SOA trials. Thus, the findings of Bryce and Bratzke (2017) cast doubt on the original explanation and motivated us to examine anew the conditions required for an introspective blind spot in the PRP paradigm using the timeline method.

In the current study, we systematically varied different factors in introspective PRP experiments to assess their impact on the introspective blind spot and to test the conscious bottleneck model. In doing so, we coincidentally also varied the sensory modalities of the two stimuli in the trial, and this emerged as an important factor in determining overall introspective accuracy on the mean data pattern. What is presented in this manuscript is the amalgamation of six experiments, each with slightly different stimuli and/or designs. All but one experiment had three SOA levels, but for simplicity in the main text these are reduced to two for all experiments. Each had a difficulty manipulation in either Task 1 or Task 2, affecting either perceptual or central processing. These manipulations and the reasons for them are reported in the relevant methods sections, but are no longer our focus in the reported results. As well as conducting the standard analysis of mean RTs and iRTs per experiment (reported in “Mean objective and introspective RTs” and Appendix A), we also analysed single trials to take advantage of the full complexity of the timeline data (reported in “Single trial analysis” and Appendix B). A cluster analysis of single trials provided insight into the types of trial recreations participants provided under different conditions, and linear mixed effect modelling allowed us to assess the relative contributions of different factors to introspective accuracy. As a preview of our findings, what emerged was a pattern whereby various factors determined introspective accuracy when S1 was auditory and S2 was visual (the typical modality order in introspective PRP experiments) but when S1 was visual and S2 was auditory, introspection was more accurate overall and unaffected by other factors. These findings pose a serious challenge to the conscious bottleneck model which is amodal in nature. As such, in the Discussion, we propose an alternative memory-based explanation for the introspective blind spot in this dual-task context in which the involvement of modality-specific subsystems can aid introspective accuracy.

## Mean objective and introspective RTs

In all six experiments reported here, trials consisted of some version of a PRP trial followed by a recreation of the trial on a timeline, and an estimate of the total trial length. The PRP trial had one auditory and one visual sub-task, both requiring button-press responses, and the difficulty of one of the tasks was manipulated (in various ways). These manipulations are reported in the relevant methods sections, but since they are no longer a particular focus, results including this factor can only be found in Appendix A. After each trial participants reported the temporal course of the trial by positioning four markers (one representing each event in the trial, S1, S2, R1 and R2) on a timeline (a horizontal line labelled ‘start’ and ‘end’) and then estimated the total trial length on a horizontal line of the same length labelled 2–6 s. A summary of defining features of the experiments is provided in Table 1. In the following, the individual methods and results for each experiment will be presented, followed by an interim summary. None of the data reported here have been previously published.

**Table 1:** Summary of experiments reported in this paper

Experiment	S1	S2	Task manipulation		Mean central gap (ms)^d^	Mean result pattern
Modality order: auditory–visual						
1A^a^	High/low tone	+ /− symbol	S2 perceptual		675	Blind spot
1B^b^	High/low tone	+ /− symbol	S2 perceptual		657	Blind spot
1C	Four tones	+ /− symbol	S1 central		663	Blind spot
Modality order: visual–auditory						
2A^﻿c^	+ /− symbol	High/low tone	S1 perceptual		717	Awareness
2B	+ /− symbol	High/low tone	S1 perceptual		644	Awareness
2C	Four digits	High/low tone	S1 central		690	Awareness

*S1* Stimulus 1; *S2* Stimulus 2

^a^1A and 1B were two conditions of one experiment, manipulated within-subjects

^b^Only S2 and R2 markers were placed by participants on the timeline. Not included in the single trial analyses

^c^Only two SOAs were included

^d^Mean central gap = the absolute ms between S2 and R1 (the two central events in a trial)

### Experiment 1A (modality order: auditory–visual)

#### Methods

Experiment 1A and Experiment 1B were two conditions of one study, manipulated within-subjects. These experiments were designed to query whether the introspective blind spot is specifically caused by dual-task demands or whether timing demands also contribute to its emergence. As such, in Experiment 1A participants were required to report all four events in the trial (note: this is a direct replication of Experiment 2A in Bryce & Bratzke, 2017), and in Experiment 1B participants were only required to report the events related to Task 2.

##### Participants

A sensitivity analysis (using G*Power; Faul et al., 2007) indicated that a sample size of *n* = 16 would give us 80% power to observe effects of SOA on iRT2 of at least *η*_*ρ*_^2^ = 0.10. Note that the comparable effect from the replay Experiment 2B in Bryce and Bratzke (2017) was considerably larger (*η*_*ρ*_^2^ = 0.50). As a much smaller effect size would still be of theoretical interest in the introspective context, this and each following experiment had 16 unique participants, recruited from the University of Tübingen.

All participants had normal or corrected vision and normal hearing, and received course credit or payment (8 € per hour). Participants were replaced if they had an error rate over 25% in the primary PRP task, if they were an outlier (more than 3 *SD*s above the mean) in terms of the percentage of grouped responses in the PRP task (grouped responses = within 100 ms of each other), or if they were an outlier in terms of the percentage of trials in which the timeline markers were not moved at all (this indicates participants were not adhering to task instructions). In Experiment 1A and 1B, two participants were replaced: one because of experimenter error, and one because of a high error rate. Of the final 16 participants, one was male and one was left-handed. The mean age of participants was 24.3 years (range 18–33 years).

##### Apparatus and stimuli

This and all subsequent experiments were conducted on a Mac computer via Matlab with the PsychToolbox extension (Brainard, 1997; Kleiner et al., 2007; Pelli, 1997), in a sound-attenuated testing booth. In Experiment 1A, stimulus 1 (S1) was a high or low frequency tone (440 or 880 Hz, 60 dB SPL) presented for 150 ms via standard (no active noise-cancelling) over-ear headphones (Sony MDR-XD200). Stimulus 2 (S2) was a white plus or minus symbol presented centrally on a black background for 500 ms. This symbol was degraded by white dots (a 1.8 × 1.8° square area of a random dot pattern). There were six levels of degradation (25 to 275 dots, in steps of 50). Responses were given via external button boxes. The duration of the trial (i.e. what the timeline would represent) was indicated by the presentation of a rectangular white frame (6.6 × 6.6°). The timeline was a horizontal line (29.5°) marked with nine evenly spaced vertical lines and labelled ‘start’ and ‘end’ at each end. Four markers represented each event in the trial: a blue circle for the first stimulus (S1), a red circle for the second stimulus (S2), a green square for left-hand response (R1), a yellow square for right-hand response (R2). A legend was presented at the top of the timeline screen to ensure participants knew what each marker represented. Participants moved the markers using computer keyboard number keys which were labelled with the same colour and shape as the corresponding markers. Total trial estimates were then given on a horizontal scale of the same length as the timeline, but marked 2–6 s.

##### Procedure and design

**Fig. 2:**
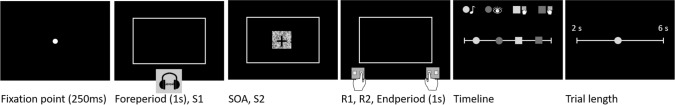
Illustration of one trial (short SOA condition) within Experiment 1A. The white frame indicates the time the timeline should represent (‘trial is on’). Participants were free to move the markers on the timeline in any order and then confirmed their final placements with the spacebar

Experiment 1A and Experiment 1B were conducted on consecutive days with order counter-balanced across participants. The trial procedure is depicted in Fig. 2. The PRP task began with a fixation point presented on the screen for 250 ms, followed by the white frame indicating the start of the trial. After a foreperiod of 1000 ms, S1 and S2 separated by the SOA (50, 250, or 1250 ms) were presented. Participants were instructed to respond as quickly and as accurately as possible to each stimulus. Responses to the tone (S1) were given with a left-hand button press (R1; low: left-hand middle finger, high: left-hand index finger) and responses to the symbol (S2) with a right-hand button press (R2; minus: right-hand index finger, plus: right-hand middle finger). After the last event in the trial there was a 1000 ms endperiod before the white frame disappeared (representing the end of the trial). In the second part of each trial, participants recreated the trial on the timeline; initially the timeline was presented with all markers in the central position. Participants were free to move each movable marker (using the relevant labelled keyboard buttons) in any order to recreate their representation of the trial events on the timeline, and pressed the spacebar to confirm the final positions of the markers. In the final part of each trial, participants then provided their total trial length estimates by moving a white marker to the left or right on the 2–6 s scale, and confirmed their estimate by pressing the spacebar. After an additional pause of 500 ms, the next trial began.

Participants completed 2 practice blocks of 18 trials each (1 with only the PRP task, and 1 with the PRP task, timeline and total trial length estimate) and 6 experimental blocks of 36 trials each. There were 72 unique trials: 2 auditory S1 (low or high tones) × 12 visual S2 (plus or minus symbols, each with 6 levels of degradation) × 3 SOA (50, 250, or 1250 ms). Each of these was presented three times, resulting in 216 experimental trials and an average running time of 1.5 h per session.

##### Planned analyses

Mean objective and introspective RT1 and RT2 were calculated and analysed for each experiment using the same method, described here. For each trial, iRT1 and iRT2 were calculated by first transforming the marker positions (in pixels) to millisecond values based on the timeline representing the objective duration of that trial, and then calculating the difference between the markers for R1 and S1, and R2 and S2, respectively (as in Bryce & Bratzke, 2017). In the interests of concision and readability, minimal results are reported from each experiment in the main text. As such, only trials from the shortest and longest SOA conditions (50 and 1250 ms) are included and analysis of the factor difficulty manipulation is not reported. Full results for each experiment can be found in Appendix A; that is, error rates, RTs, and iRTs as a function of SOA (all levels) and difficulty manipulation. Reported in the main text are the results of an omnibus ANOVA to determine the awareness of the PRP effect. Awareness is defined as a larger SOA effect on iRT2 than on iRT1; as such iRTs were analysed with the within-subjects factors of SOA (50 or 1250 ms) and Task (1 or 2). Previous studies have defined the unawareness of the PRP effect as the absence of a SOA main effect on iRT2. We consider this an inappropriate measure as a similarly sized SOA effect on both iRT1 and iRT2 would be mistakenly defined as introspective ‘awareness’ of the PRP effect even though such a data pattern would not reflect accurate introspection in this context. The measure used here improves upon previous approaches by ensuring that the label of ‘awareness’ is only granted when participants’ introspective reports indicate they are aware of a greater performance cost in their RT2 than RT1. The Greenhouse–Geisser correction was used to adjust *p* values where appropriate and partial Eta-squared effect sizes are provided. Standard errors for within-subjects designs were calculated according to Morey (2008).

#### Results

**Fig. 3:**
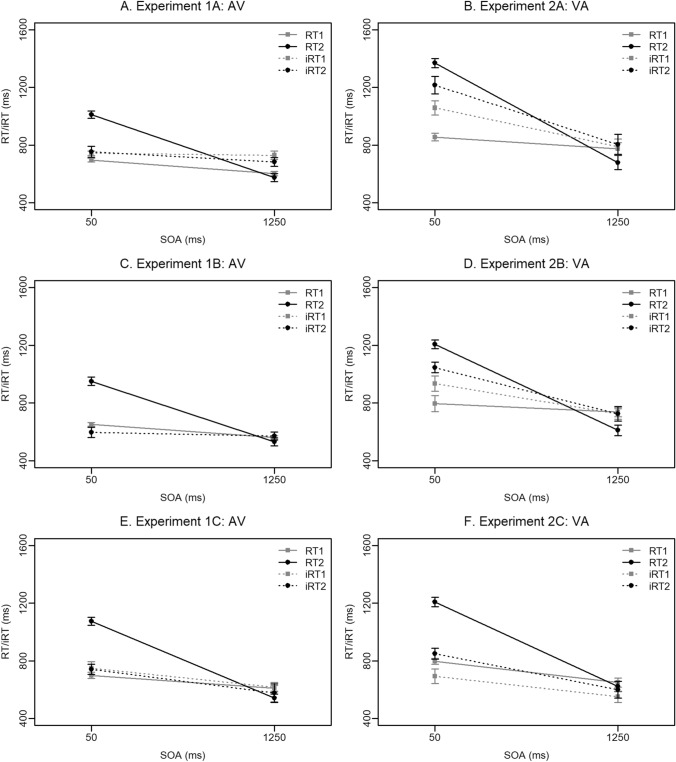
Mean RT1 and RT2 (solid grey and black lines, respectively) and iRT1 and iRT2 (dashed grey and black lines, respectively) as a function of stimulus onset asynchrony (SOA) for each experiment. On the left-hand column are all the experiments in which S1 was auditory and S2 was visual (modality order: AV). On the right-hand column are the experiments in which S1 was visual and S2 was auditory (Modality order: VA). Error bars represent ± 1 within-subjects *SE*. Note: iRT1 is not plotted for Experiment 1B since participants only placed the markers relating to Task 2 on the timeline

Some trials were excluded before the data were analysed: those with errors in either of the PRP sub-tasks (12.07%), those in which RT1 or RT2 deviated more than three standard deviations from the individual mean in each condition (2.67% of correct trials), and those in which the inter-response interval was less than 100 ms (this included grouped responses and when R2 was provided before R1; 0.30% of remaining trials). Mean reaction times (RTs) and introspective reaction times (iRTs) for Task 1 and Task 2 are presented in Fig. 3A. The SOA × Task ANOVA on iRTs indicated an introspective blind spot, as the SOA × Task interaction was not significant, *F*(1,15) = 2.24, *p* = 0.155, *η*_*p*_^2^ = 0.13.

Consistent with the results of Experiment 2A in Bryce and Bratzke (2017), and all other introspective PRP experiments published to date, participants in this experiment with an auditory S1 and visual S2 seemed to suffer from an introspective blind spot.

### Experiment 1B (modality order: auditory–visual)

#### Methods

##### Participants

See “Participants” for Experiment 1A for participant information.

##### Apparatus and stimuli

See “Apparatus and stimuli” for Experiment 1A.

##### Procedure and design

The procedure was mostly the same as described in “Procedure and design” for Experiment 1A, except that in Experiment 1B participants were only required to place the markers relating to Task 2. As such, when the timeline was presented the markers representing S1 and R1 were already correctly fixed in place on the timeline with a tick mark (✓) above them.

#### Results

Before analysis some trials were rejected according to the same criteria named in “Results” of Experiment 1A. In this case this amounted to 10.42% due to errors, a further 2.36% due to RT outliers, and a further 0.30% due to response grouping or reversals. Mean RT1, RT2, and iRT2 are presented in Fig. 3C (iRT1 is not plotted as participants only placed markers relating to Task 2). The SOA × Task ANOVA (which in this case included the objective RT1 instead of iRT1) indicated an introspective blind spot, as the SOA × Task interaction was not significant, *F*(1,15) = 2.56, *p* = 0.130, *η*_*p*_^2^ = 0.15.

The aim of Experiment 1B was to investigate whether timing demands contribute to the introspective blind spot. The data pattern indicates that they do not, as even when participants were required only to place markers relating to Task 2, they did not report the PRP effect.

### Experiment 1C (modality order: auditory–visual)

Experiment 1C was designed to test a crucial assumption of the conscious bottleneck model, that perception of the second stimulus is tightly linked to the end of Task 1 central processing. To this end, the end of Task 1 central processing was varied via a central stage difficulty manipulation.

#### Methods

##### Participants

One participant had to be replaced because of a high error rate. Of the final 16 participants, 3 were male and all 16 were right-handed. The mean age of participants was 24.3 years (range 20–28 years).

##### Apparatus and stimuli

S1 was one of four auditory stimuli - tones with frequencies 400, 660, 1020, and 1400 Hz^1^ to-be-compared to a reference tone of 800 Hz. All tones had a duration of 150 ms and an amplitude of 60 dB. S2 was a simple plus or minus symbol presented in the centre of the screen. All other stimuli and apparatus were the same as previously described.

##### Procedure and design

The reference tone was presented to participants during the presentation of the fixation point. Task 1 was to categorise the frequency of S1 as lower (left-hand middle finger button press) or higher (left-hand index finger button press) than the reference tone. The two frequencies closest to the reference tone (660 and 1020 Hz) comprised the ‘hard’ condition and those further from it (400 and 1400 Hz) comprised the ‘easy’ condition. Task 2 was to respond to the visual stimulus: a right-hand index finger button press in response to the minus symbol and a middle finger button press in response to the plus symbol. In this experiment, there were 24 unique trials (4 S1 tones × 2 S2 symbols × 3 SOAs), each repeated nine times to maintain the same number of experimental trials and running time as the other experiments. All other procedural details were the same as previously described.

#### Results

In this dataset, 13.22% of trials were removed due to errors, a further 2.89% were removed due to outlier RTs, and an additional 0.36% because of grouped responses. Mean RTs and iRTs are presented in Fig. 3E. The SOA × Task ANOVA indicated an introspective blind spot, as the SOA × Task interaction was not significant, *F*(1,15) = 0.43, *p* = 0.521, *η*_*p*_^2^ = 0.03.

Experiment 1C tested an assumption of the conscious bottleneck model, that prolonging Task 1 central processing would delay the conscious awareness of S2 and, therefore, enhance the introspective blind spot. Consistent with this, an introspective blind spot was observed in the mean data. However, there was no evidence that the placement of the S2 marker was linked to the end of Task 1 central processing, as there was no significant effect of Task 1 difficulty on the S2 marker position, *F*(1,15) = 1.15, *p* = 0.300, *η*_*p*_^2^ = 0.07, nor on the time this represented in ms, *F*(1,15) = 1.01, *p* = 0.917, *η*_*p*_^2^ < 0.01. As such, the results of this experimental manipulation are not in line with the conscious bottleneck model.

### Experiment 2A (modality order: visual–auditory)

#### Methods

The aim of Experiment 2A was the same as for Experiment 1C- to delay the end of Task 1 central processing - but this time with the alternative modality order. This was achieved by a perceptual degradation of the visual S1.

##### Participants

Three participants were replaced: one because of a high error rate, one due to response grouping in 43% of PRP trials, and one because they did not move the timeline markers on 37% of trials. Of the final 16 participants, four were male and all were right-handed. The mean age of participants was 23.7 years (range 18–35 years).

##### Apparatus and stimuli

Testing conditions and features of the visual and auditory stimuli were as described for Experiment 1A. In this experiment, the symbol (plus or minus) was presented as S1 and the tone (440 or 880 Hz) was presented as S2.

##### Procedure and design

In this experiment, S1 was a symbol degraded by a random dot pattern (responded to with the left-hand) and S2 was the tone (responded to with the right-hand). As it was originally intended to analyse the six levels of Task 1 degradation in three levels (low, medium and high degradation), the number of SOAs was reduced to two (50 and 1250 ms). There were 48 unique trials (12 S1 symbols × 2 S2 tones × 2 SOAs), each repeated 6 times to make up the experimental trials. The participants experienced this as 12 blocks of 24 trials and the experiment had an average running time of 1.5 h. Other aspects of the procedure and design were as previously described.

#### Results

A total of 14.22% of trials were removed due to errors, an additional 3.22% due to outlier RTs, and a further 0.38% because of grouped or reversed responses. Mean RTs and iRTs are presented in Fig. 3B. The SOA × Task ANOVA on iRTs indicated an awareness of the PRP effect, as the SOA × Task interaction was significant, *F*(1,15) = 5.68, *p* = 0.031, *η*_*p*_^2^ = 0.27.

Experiment 2A was conducted to examine the effect of delaying the end of Task 1 central processing on the introspective blind spot, but this time in an experiment with a visual-auditory modality order. In contrast to the results of Experiment 1C, the mean data indicated an awareness of the PRP effect and as such these findings are also not in line with the conscious bottleneck model.

### Experiment 2B (modality order: visual–auditory)

Experiment 2B was a replication of Experiment 2A with three SOA levels included, conducted to check whether reducing the number of SOA levels to two had caused awareness of the PRP effect in Experiment 2A.

#### Methods

##### Participants

Of the 16 participants, 5 were male and 15 were right-handed. The mean age of participants was 22.9 years (range 19–30 years).

##### Apparatus and stimuli

Testing conditions and stimuli are as described in “Apparatus and stimuli” of Experiment 2A.

##### Procedure and design

The trial procedure was the same as described in “Procedure and design” of Experiment 2A. The only change was the levels of SOA (they were again 50, 250 and 1250 ms) and the number of repetitions per trial. Since there were now three levels of SOA, we planned to analyse the six levels of Task 1 degradation in two levels (low and high degradation). There were 72 unique trials (12 S1 symbols × 2 S2 tones × 3 SOAs), each repeated three times to make up the experimental trials. The participants experienced this as 6 blocks of 36 trials and the experiment had an average running time of 1.5 h.

#### Results

As before, trials with errors were removed (13.77%), as were trials with an RT outlier (2.75% of correct trials), and trials with grouped or reversed responses (a further 1.68%). Mean RTs and iRTs are presented in Fig. 3D. The SOA × Task ANOVA on iRTs indicated an awareness of the PRP effect, as the SOA × Task interaction was significant, *F*(1,15) = 6.45, *p* = 0.023, *η*_*p*_^2^ = 0.30.

Experiment 2B was conducted to investigate whether reducing the number of SOAs included in Experiment 2A could have led to the unexpected awareness of the PRP effect in that experiment. A similar awareness was observed in Experiment 2B, suggesting that the number of SOAs included in the experimental design does not contribute to the awareness of the PRP effect.

### Experiment 2C (modality order: visual–auditory)

With Experiment 2C we aimed to delay the end of Task 1 central processing via a direct manipulation of the length of central processing, as opposed to via a perceptual manipulation as in Experiments 2A and 2B. The motivation for this was to test an alternative explanation for the awareness of the PRP effect in Experiments 2A and 2B, namely that S2 could be more accurately reported at short SOAs because its presentation coincided with perceptual processing of Task 1 rather than central processing.

#### Methods

##### Participants

One participant had to be replaced in this experiment due to grouped responding in the PRP task on 33% of trials. Of the 16 final participants, 5 were male and 13 were right-handed. The mean age of participants was 22.4 years (range 19–33 years).

##### Apparatus and stimuli

Testing conditions and most stimuli were as previously described. The only exception was the visual S1. In this experiment, S1 was a number (28, 37, 53, 62) presented in white on a black screen in Arial font, size 24.

##### Procedure and design

Task 1 was to indicate whether the number presented as S1 had a greater or smaller value than 45; 28 and 62 were classified as ‘low’ and 37 and 53 as ‘high’ difficulty. In this experiment, there were 24 unique trials (4 S1 numbers × 2 S2 tones × 3 SOAs), each repeated nine times to maintain the same number of experimental trials and running time as previous experiments.

#### Results

Here participants made an error in 10.65% of trials and these were removed, 3.13% of trials were removed due to outlier RTs, and 0.71% due to grouped or reversed responses. Mean RTs and iRTs are presented in Fig. 3F. The SOA × Task ANOVA on iRTs indicated an awareness of the PRP effect, as the SOA × Task interaction was significant, *F*(1,15) = 13.56, *p* = 0.002, *η*_*p*_^2^ = 0.47.

In Experiment 2C the end of Task 1 central processing was delayed directly via a central stage manipulation. This experiment was designed to address an alternative explanation for the awareness of the PRP effect in Experiments 2A and 2B, that S2 could be consciously perceived at short SOAs because its presentation coincided with perceptual processing of Task 1 rather than central processing. As the data pattern indicated awareness of the PRP effect, this alternative explanation can be rejected.

### Interim discussion

A rather clear data pattern can be seen in Fig. 3 - experiments with a modality order AV (left column, Experiments 1A–C) seem to produce introspective blind spots, whereas experiments with a modality order VA (right column, Experiments 2A–C) seem to produce more accurate introspective reports. The results of Experiments 1A–C are consistent with the results of previously published introspective PRP studies that used a modality order AV (Corallo et al., 2008; Marti et al., 2010; Experiment 1 of Bryce & Bratzke, 2014; Bratzke & Bryce, 2016; Experiment 2a of Bryce & Bratzke, 2017, Experiment 1 of Klein, 2015). In addition, which task was manipulated and exactly how this was done does not seem to affect introspection in this context.

The surprising and novel finding that in three experiments (2A–C) participants could rather accurately report a larger effect of SOA on RT2 than RT1 contributes substantially to our understanding of the nature of the introspective blind spot. These results suggest that a blind spot does not necessarily emerge every time the central processing bottleneck is engaged, providing strong evidence against the conscious bottleneck model. Instead, this overall data pattern nudged us in the direction of considering other aspects of the trial that may influence introspective accuracy.

As well as having an AV modality order, the experiments in which an introspective blind spot was observed seemed to have slightly longer RTs overall (see Fig. 3) and a slightly longer central gap (see Table 1). That is, the two central events in the trial occur on average 665 ms apart in Experiments 1A–C, and on average 684 ms apart in Experiments 2A–C. Although a small difference, we hypothesised that the time in the centre of the trial may contribute to the accuracy of introspective reports because of the important role of time in some models of working memory (e.g. the Time-based Resource-sharing model of Barrouillet et al., 2004). It is hard to assess the relative contribution of modality order and central gap to the presence or absence of an introspective blind spot from the mean data patterns. Instead, a linear mixed effects model that makes use of single trial data has the potential to pull these contributions apart.

## Single trial analysis

To more sensitively quantify the degree of introspective accuracy in a single trial and to be able to address the question ‘what contributes to introspective accuracy in the PRP context?’ we conducted a further analysis on the level of single trials. All trials from the shortest and longest SOA conditions (50 and 1250 ms) of experiments 1A, 1C, 2A, 2B and 2C were included in this analysis (Experiment 1B was not included as the timeline marker positions for S1 and R1 were not placed by the participant). A measure of introspective accuracy was calculated for each trial (described below) and a linear mixed effects model was fitted to these data. This model examined the role of three predictors (modality order, SOA, the central gap; elaborated upon below) in determining introspective accuracy in a trial.

### Methods

To understand how we derived a measure of introspective accuracy for each trial, we have to conceptualise a PRP trial not as comprising two RTs, but four separate events (S1, S2, R1, R2). Likewise, the participant’s recreation of the trial on the timeline comprises four separate markers. Introspective accuracy in a trial is determined by how similarly the temporal structure of the timeline recreation matches the temporal structure of the trial. That is, our measure takes into account the relationships between all four events or markers, rather than just RTs. We opted to use this more complex approach of considering the positions of all four events / markers rather than the ‘difference’ measures of RTs/iRTs as we also aimed to determine what sort of trial structures participants recreated on the timeline when their introspections were not accurate.

**Fig. 4:**
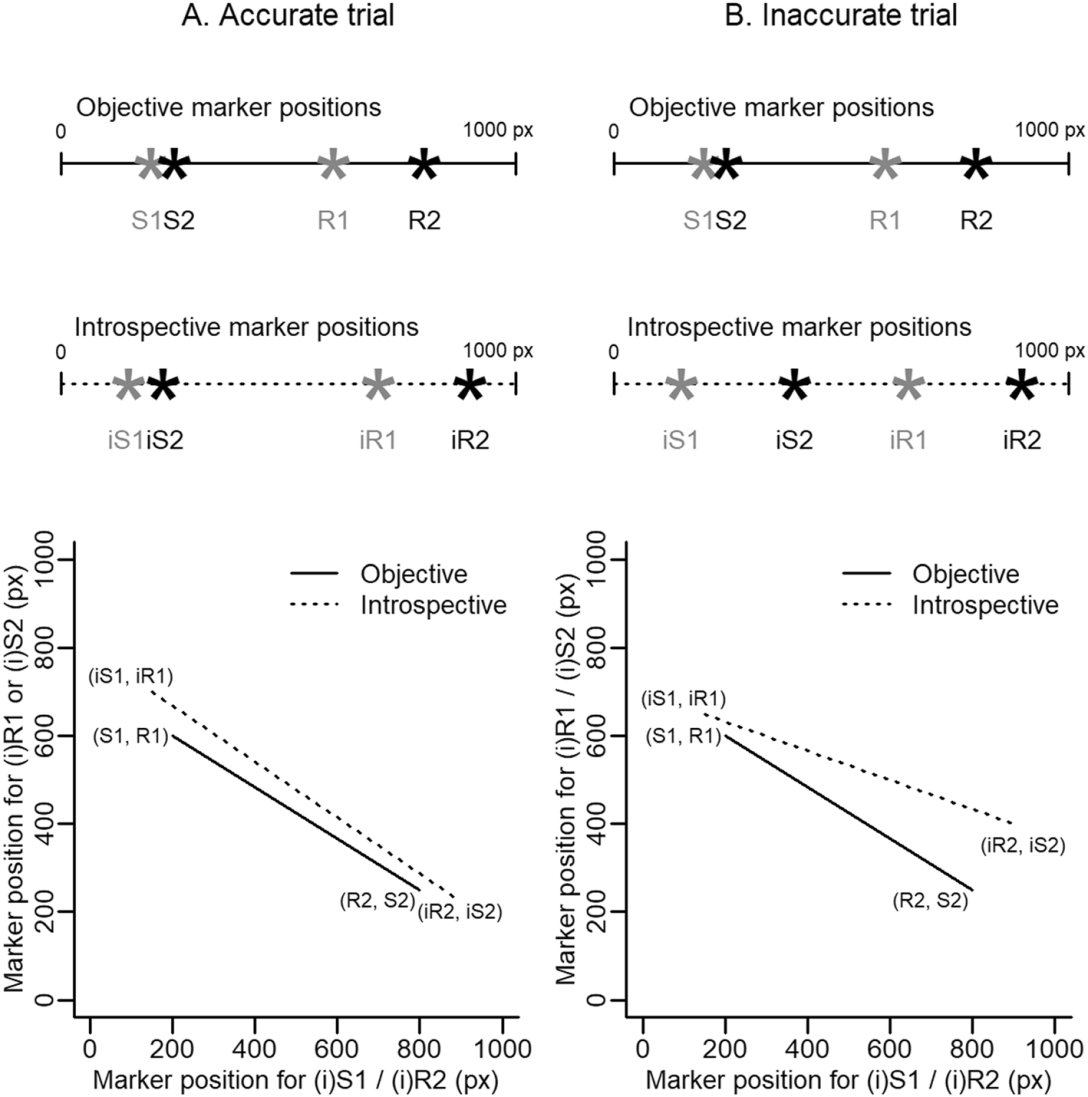
Illustration of two example trials and the corresponding lines from which the radians were calculated. The lines are drawn based on the corresponding marker position (in pixels) on the screen through (i/S1, i/R1) and (i/R2, i/S2). Panel A illustrates a rather accurate trial; the participant has placed the markers in a similar pattern as the objective timing of events. As such, their radians are similar (lines almost parallel; rad_obj_ = − 0.53 rad_intro_ = − 0.56). Panel B illustrates a rather inaccurate trial; although the markers are placed in the correct order they are evenly spaced across the timeline. Accordingly, their radians deviate more from one another (rad_obj_ = − 0.53 rad_intro_ = − 0.32). Note: S1 = stimulus 1; S2 = stimulus 2; R1 = response to the first task; R2 =response to the second task; i = the introspective marker position

We derived the measure in the following way. The events in a trial (S1, S2, R1, R2) or the markers on a timeline (iS1, iS2, iR1, iR2) can be converted to a line and the radian of that line’s slope is used as a summary value for the temporal structure of the trial or timeline, respectively (see Fig. 4 for an illustration of this method). That is, to calculate the introspective radian (rad_intro_) the marker positions on the timeline (i.e. the pixel values on the screen) were used to plot a line through (iS1, iR1) and (iR2, iS2), and the radian of the slope calculated according to the formula:

$${\text{rad}}_{{{\text{intro}}}} = {\text{tan}}^{{ - 1}} \frac{{{\text{iS}}2~{-}~{\text{iR}}1}}{{{\text{iR}}2~{-}~{\text{iS}}1}}$$

For 20 trials (0.18% of all trials) rad_intro_ could not be calculated as all markers had the same position; these were removed from further analysis. To calculate the objective radian (rad_obj_), the timing of events in the trial (in ms) were first converted to marker positions on the timeline (as if we were to make a graphical representation of the trial), a line plotted and the radian of the slope calculated as above. If the markers were placed quite accurately in a trial, the objective and introspective lines were well aligned and parallel, and the difference between the radians was small (see Fig. 4A). If, however, the markers were placed in a different order or with different temporal structure than they really occurred, the objective and introspective lines did not align, and the difference between the radians increased (see Fig. 4B).

A cluster analysis was carried out to assess the validity of using these radians to summarise the (objective or introspective) temporal structure of a trial and to explore the types of trial structures participants recreated on the timeline. Details of this analysis and an outline of the findings can be found in Appendix B. In summary, this analysis confirmed that the radians are interpretable and informative.^2^ Indeed, the difference between the objective radian and the introspective radian in a trial provided a good measure of the accuracy of introspection in that trial. Accordingly, the absolute difference in radians (∆rad =|rad_obj_ − rad_intro_|) was calculated for each trial and used as the dependent variable for the linear mixed effects (LME) model analysis.

**Fig. 5:**
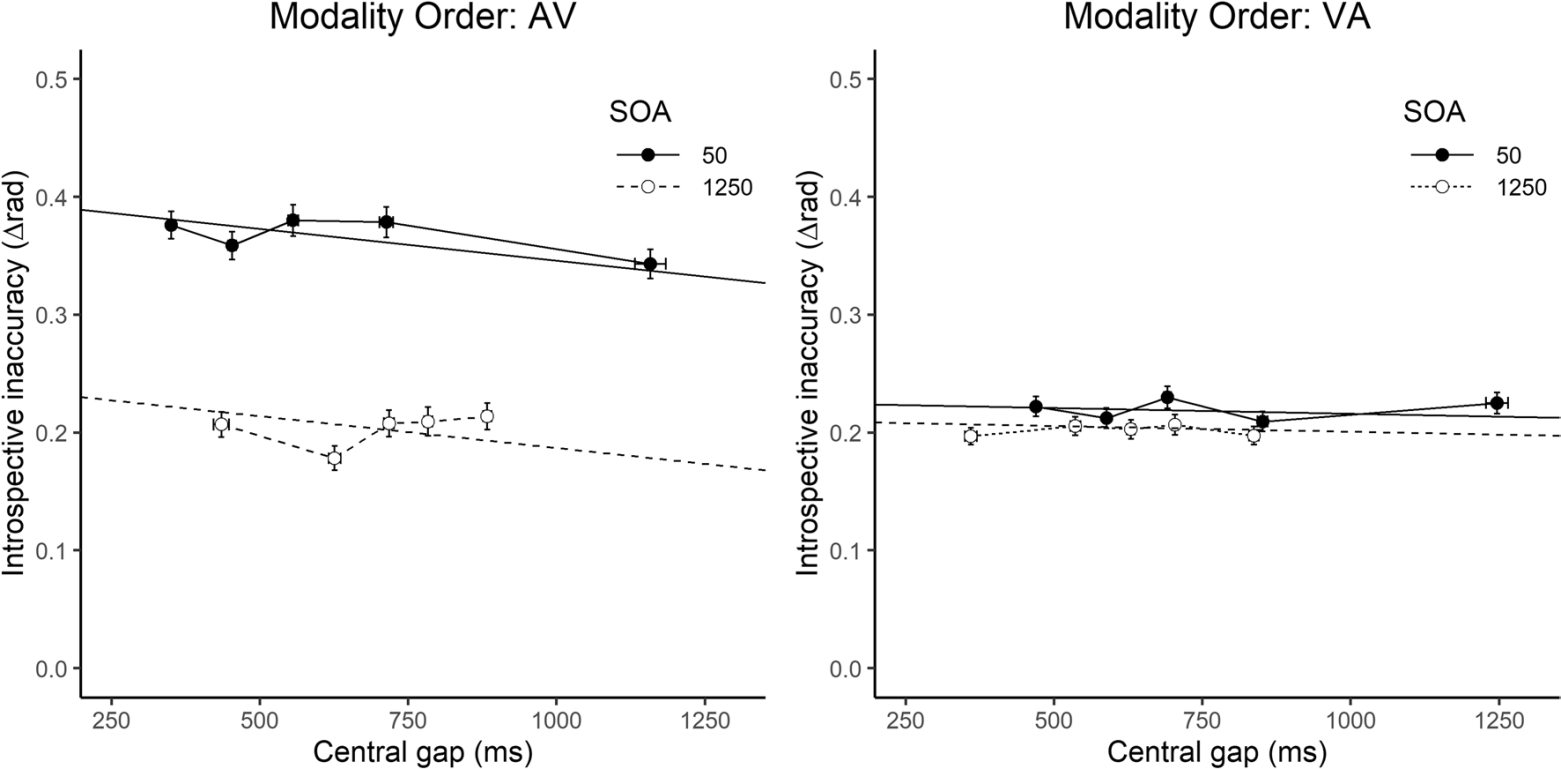
Vincentized data from five experiments combined (Experiments 1A, 1C, 2A, 2B, 2C). Introspective inaccuracy (∆rad) is plotted against the central gap (in ms) as a function of stimulus onset asynchrony (SOA; 50 or 1250 ms) and modality order (AV or VA). Single trials were vincentized based on the central gap. That is, the first vincentile in each panel represents the 20% smallest central gap trials for each participant by SOA; the second contains the next 20% of smallest central gap trials, and so on. Error bars represent ± 1 within-subject *SE*. Non-transformed variables were used for plotting. Regression lines for fixed effects are plotted. Note: AV = S1 is auditory, S2 is visual; VA = S1 is visual, S2 is auditory; Solid lines and black circles = 50 ms SOA; Dashed lines and white circles = 1250 ms SOA

The aim of the LME model was to determine what predicts introspective (in)accuracy (∆rad) in a trial from three predictor variables - modality order (AV or VA), SOA (50 or 1250), the central gap in the trial. The central gap was calculated as the absolute difference in milliseconds between the central two events in the trial, S2 and R1. These typically occur in a different order depending on SOA condition: in the short SOA condition a typical event order is S1–S2–R1–R2, in the long SOA condition a typical event order is S1–R1–S2–R2. Since we did not want central gap to be confounded with SOA and we hypothesised that the time available in the centre of the trial may contribute to introspective accuracy, we included the absolute gap in our model rather than a measure of task overlap. Modality order is a second order factor, as this was manipulated between-subjects. Main effects and all two-way interactions were entered as fixed effects into the full model, and the random effects structure allowed intercepts and slopes to vary per participant and SOA (using the R package lmerTest; Kuznetsova et al., 2017). A process of model selection was then conducted whereby likelihood ratio tests assessed the significance of fixed effects by comparing each reduced model with the more complex model. Transforming the continuous variables (∆rad and central gap, with a square-root transformation) resulted in residuals that were more normally distributed than when raw values were entered in the model. As such, transformed values were entered into the LME model, whereas raw values are depicted in Fig. 5.

### Results

Linear mixed effects model analysis with ∆rad (a measure of *introspective inaccuracy*) as the dependent variable revealed significant main effects of Modality Order, χ^2^(1) = 8.65, *p* = 0.003, SOA, χ^2^(1) = 8.52, *p* = 0.004, and central gap, χ^2^(1) = 20.73, *p* < 0.001 (estimates of the final model are in Table 2). There were also significant interactions between modality order and SOA, χ^2^(1) = 8.53, *p* = 0.003, and modality order and central gap, χ^2^(1) = 11.32, *p* < 0.001. The interaction between SOA and central gap was not significant, χ^2^(1) = 0.34, *p* = 0.558. The inclusion of the random effects structure in which intercepts and slopes could vary per participant and SOA significantly improved the model, χ^2^(3) = 3870.30, *p* < 0.001, indicating some intra-individual variability in the effect that the predictors have on introspective accuracy.^3^

**Table 2:** Estimates for fixed effects in the best fitting linear mixed effects model with introspective inaccuracy (∆rad) as dependent variable

Predictor	Estimate	*SE*	95% CI	*p* value
Intercept	0.628	0.029	[0.57, 0.68]	*p* < 0.001
Modality order^a^	− 0.202	0.037	[− 0.27, − 0.13]	*p* < 0.001
SOA^b^	− 0.149	0.035	[− 0.22, − 0.08]	*p* < 0.001
Central gap	− 0.003	0.0006	[− 0.004, − 0.002]	*p* < 0.001
Modality order × SOA	0.136	0.045	[0.05, 0.22]	*p* = 0.004
Modality order × central gap	0.002	0.0007	[0.001, 0.004]	*p* < 0.001

∆rad and Central gap were square-root transformed for the LME model. *p* values are based on the Satterthwaite approximation

^a^Baseline: AV

^b^Baseline: 50 ms SOA

Introspective accuracy is overall better in the VA condition than the AV condition and in trials with long than short SOAs. Participants’ introspective reports become more accurate as the gap in the centre of the trial increases. The interactions indicate different effects of SOA and the central gap on introspective accuracy depending on the modality order, as can be seen in Fig. 5. That is, in the AV condition, SOA and the central gap seem to contribute to the accuracy of introspective reports, but in the VA condition they have a minimal or no effect. More specifically, in the AV condition introspective reports are more accurate when the SOA is long, and when the central gap is longer.

## Discussion

A series of introspective PRP experiments were conducted to identify the determinants of introspective accuracy. To our surprise, the sensory modality of the two stimuli within the trial emerged as an important predictor of whether the mean data pattern indicated an introspective blind spot or not. Subsequent single trial analyses provided an insight into the variety of temporal structures recreated by participants on the timeline. The first important empirical contribution of this study is that the data are inconsistent with the conscious bottleneck model, as S2 did not seem to be specifically misperceived in short SOA trials. A linear mixed effects model provided more clarity regarding the contribution that different measurable factors make to introspective accuracy in PRP trials. That is, introspective reports were more accurate in the visual-auditory modality order than the auditory-visual modality order, and in the auditory-visual modality order other temporal gaps in the trial made significant contributions to introspective accuracy. Overall, this series of experiments provided novel insights into introspection during dual-task processing and highlighted an important role for memory in introspection in such attentionally demanding contexts.

The majority of introspective PRP experiments reported in the literature to date have used an auditory-visual modality order. As such, they observed an introspective blind spot and the predominant explanation for the cause of the blind spot (namely, the conscious bottleneck model) remained unchallenged, regardless of the method used to collect introspective reports. Aside from two exceptions (elaborated on below), it is safe to say that the series of experiments presented here constitutes the most consistent evidence of participants being aware of their dual-task costs. The very observation of awareness of the PRP effect in three separate experiments poses a serious challenge to the conscious bottleneck model. The idea that a second stimulus cannot reach conscious awareness while central processing of the first stimulus is ongoing represents a structural limitation of consciousness; as such, the modality of these stimuli should not play a role in its occurrence.

The cluster analysis identified a variety of reported temporal structures across five experiments. Within the short SOA trials, only one cluster (Cluster 7, see Appendix B) can be interpreted as being consistent with the conscious bottleneck model, as it appears that the S2 marker is temporally tied to R1 (a proxy for end of Task 1 central processing). It is important to caution against interpreting the size of clusters too strongly, since this is highly influenced by how many clusters are sought in the K-means method and the cluster analysis was exploratory in nature. However, even if this pattern of timeline recreation can be attributed to the conscious bottleneck model, this is rather strong evidence against a consciousness bottleneck being the sole or dominant reason for poor introspective accuracy in the PRP task. In terms of clusters deemed ‘inaccurate’, various biases seem to be at play and there appears to be no single systematic way in which participants misrepresent the temporal structure of trials. If the conscious bottleneck model were true, we would expect the pattern produced in Cluster 7 to be much more dominant in the dataset and for the majority of long SOA trials to be accurately reported. Additionally, all clusters seem to be composed of trials from both AV and VA modality orders. Thus, a modality-specific version of the conscious bottleneck in which the conscious bottleneck only occurs in the AV modality is also inconsistent with these data.

It is evident from the linear mixed effects model that modality order has a clear impact on the accuracy of introspection in these experiments. The model indicated not only that introspective accuracy is overall better in the VA than the AV modality order, but that the influence of other predictors is also affected by modality order. In AV experiments, other temporal gaps in the trial (such as the central gap and the SOA) affect introspective accuracy, whereas in VA experiments none of the variables entered into the model seem to have a substantial effect on introspective accuracy. Although the data patterns appear to greatly differ across modality orders, we propose that the memory processes available during a PRP trial can account for both. This idea developed from our observation that the events in a PRP trial may be experienced as a sequence or rhythm with a particular temporal structure, and that such a sequence would be experienced differently in each modality order. That is, trials with a VA modality order can be conceptualised as a visual event followed by three auditory events, as the two button presses also produce clear auditory events (“clicks” when buttons are pressed). This sequence of events could be experienced as an auditory rhythm. The AV modality order, however, has no such auditory rhythm as the visual stimulus occurs as either the second or the third event in the trial.

In the VA modality order, we assume that the identity of the three auditory events (S2, R1, R2) as well as the temporal structure of the sequence they comprise can be stored and rehearsed rather efficiently in working memory, perhaps via a modality-specific subsystem such as the phonological loop in Baddeley’s (1986) model. Although Saito’s (2001) individual differences approach suggests that the articulatory rehearsal component does not support memory for rhythms, he still situates the encoding and maintenance of auditory rhythms within the phonological loop. Relevant findings from the field of memory indicate an auditory advantage for sequences of stimuli (Collier & Logan, 2000). In a same-different judgement task, participants performed better when both sequences were auditory than when one or both were presented in the visual modality. The authors speculated that when comparing sequences of different modalities, participants may have to rely on a common amodal code or may have to convert all sequences into the auditory modality (see also Bratzke & Ulrich, 2019; Guttmann et al., 2005) Accordingly, we propose that in AV trials when trial information composed of different modalities has to be stored, encoding and rehearsal cannot be easily achieved in a modality-specific subsystem and would, therefore, benefit from more time between events. Indeed, within the field of working memory, some theorists have proposed that processing in a primary task (here, the PRP task) can prevent or compete with the reactivation of memory traces (Barrouillet et al., 2004; Towse et al., 2000). Barrouillet and colleagues refer to this as a bottleneck for retrieval activities that demand a rapid and frequent switching between refreshing memory traces and task processing. That such rapid switching may be limited in an introspective PRP context is supported by the observation that SOA and central gap are significant predictors of introspective accuracy in the AV modality order.

Another memory process may also contribute to the superior introspective accuracy when the trial events can be represented as a sequence of auditory stimuli, namely sensory memory. This is a relatively short-lived modality-specific memory trace which is longer for auditory than for visual stimulation (e.g. Darwin et al., 1972) and does not require any attentional resources or rehearsal processes. Given that the total duration of PRP trials in this dataset (including 1 s fore- and end-periods) falls into the range of previous estimates of the duration of auditory sensory memory (about 2–10 s; e.g.,Nees, 2016; Sams et al., 1993), participants may rely more strongly on sensory memory when recreating VA than when recreating AV trials. However, two features of the current data suggest that sensory memory alone cannot account for the current results. First, we have evidence of accurate introspection in many AV trials where the modality-specific sensory memory cannot be the only process involved. As can be seen in Table 9 in Appendix B, 51% of trials from AV experiments belong to clusters characterised by ‘accurate’ or ‘mostly accurate’ introspection. We hypothesise that working memory was engaged in those contexts, and other trial-by-trial fluctuations in cognitive resources and time within the trial determined introspective accuracy (an account supported by the LME model results). Second, a sensory memory account would predict better introspective accuracy when trials are shorter as the memory trace would be stronger at the point of timeline recreation. Our data indicate just the opposite introspective accuracy in AV experiments was better when the SOA was long and the central gap was longer (both positively correlated with the total trial duration). Based on these observations, we posit that working memory plays a crucial role in introspection in the PRP context.

The memory account proposed here makes testable predictions about the factors that determine introspective accuracy. For example, a PRP task with two auditory stimuli should result in even more accurate introspective reports and, conversely, the dampening of auditory feedback (e.g. using noise-cancelling headphones) should result in less accurate introspective reports. Increasing time in the centre of the trial via other manipulations should also improve introspective accuracy. Furthermore, altering the working memory storage demands of a task should affect the accuracy of introspective reports, for instance by simplifying stimulus–response rules. Another interesting and possibly related finding from the field of dual-task processing is the effect of input–output modality compatibility on dual-task costs (i.e. smaller dual-task costs for auditory-verbal and visual-manual tasks compared with auditory-manual and visual-verbal tasks; e.g. Hazeltine et al., 2006). Reminiscent of the memory account of introspection proposed here, Hazeltine and Wifall (2011) attributed these modality-pairing effects to the involvement of modality-specific subsystems of working memory. Schacherer and Hazeltine (2020) even observed that dual-task costs were smaller when immediate modality-compatible action effects accompanied a response (e.g. presentation of the word “dog” when the participant responded to a dog’s bark) than when no additional action effects were presented. One could speculate that such effects on performance may be driven by improved introspective accuracy; the addition of action effects may have emphasised the temporal structure of the trial, leading to more successful encoding of it (i.e. more accurate introspection), allowing the participant to adjust their strategy and/or resource investment and, therefore, improve their performance. Such avenues of research could shed light on the contexts that result in a failure to successfully encode and rehearse information about the temporal structure of the trial, and thus further specify this memory account.

Two previous cases of awareness of the PRP effect can now be reconsidered from the perspective that memory processes play a key role in introspective accuracy here. In an earlier publication of ours (Bryce & Bratzke, 2014) we observed awareness in an experiment with a visual S1 (a plus/minus symbol) and an auditory S2 (a high/low tone) just as in the current Experiment 2B. However, we (perhaps mistakenly) attributed this apparent awareness to an accompanying greater feeling of difficulty in short than long SOA trials (supported by the fact that we also observed higher error rates in the short than the long SOA condition). With the insights afforded by these new data, we propose that memory processes can also account for that result. Bratzke and Janczyk (2021) also observed an awareness of the PRP effect in their Experiment 1 in which participants responded to a visual S1 (a H or S) with a hand response, and to a visual S2 (red or green colour change) with a foot response. It is possible that the inclusion of only one stimulus modality (i.e. visual) and one response modality (i.e. manual) sufficiently simplified memory encoding resulting in improved introspective accuracy in this context. Alternatively, perhaps the memory processes we discuss here do not play such a prominent role in introspective reports given via visual analogue scales as in these studies; indeed, such estimates may be largely cue-based as described in the cue-utilisation view of metacognitive monitoring (e.g. Koriat, 2012). Such cues may include feelings of difficulty or other temporal intervals in the trial (for similar arguments see Bryce & Bratzke, 2014; Bratzke et al., 2014).

Having attributed differences in introspective accuracy to the interplay between task processing and memory encoding, the question arises, is this task really assessing introspection or simply memory for events and their timing? We would posit that the recreation of a trial on a timeline is not equivalent to a pure memory task, as these events have meaning to the participants. They must report stimuli that they have processed and their own responses, rather than four independent stimuli. Further, while superficially similar, we do not consider this introspective context to be comparable to classic cross-modal timing tasks (such as Azari et al., 2020; Bratzke & Ulrich, 2019; Bryce et al., 2015), since the to-be-judged time intervals are filled with cognitive processing. Empirical evidence for the introspective context being qualitatively different from either a memory task or a cross-modal timing task is provided by a previous study of ours (the ‘replay’ context of Experiment 2 in Bryce & Bratzke, 2017). In that study, participants had to report the events in a PRP trial that someone else had processed, that is as a passive observer; we observed rather accurate timeline recreations even with an AV modality order. As such, we posit that reporting a PRP trial after having processed it is qualitatively different from reporting the events as a passive observer (see also Klein & Stolz, 2018). The question remains, however, whether the introspective reports assessed here are comparable to what is commonly referred to as introspection or metacognitive monitoring. Indeed, recreating the time-course of a trial is different from providing confidence judgements or judgements of learning, the more common methods applied in metacognition research. The degree to which recreated timeline reports may be consistent with or contradict more typical monitoring judgements remains to be established within the same study. Another interesting empirical question that comes from the metacognition literature is whether these local judgements (made trial-by-trial) would be consistent with global judgements (given at the end of an experiment) in this context, or whether different sources of information may contribute to each type of judgement (as found, for instance, by Händel et al., 2020).

While the single trial approach taken here has revealed novel insights into what contributes to introspective accuracy in the PRP paradigm, it is not without limitations. The first limitation concerns the loss of (potentially important) information within each individual experiment when one opts to combine data from multiple experiments. To address this we have provided detailed results of each experiment in Appendix A for the interested reader. Another limitation concerns the LME analysis, in which only certain measurable variables are entered as predictors. It is possible that other factors we did not measure, are inherently unmeasurable, or have not considered may be important predictors of introspective accuracy. Finally, the fact that modality order was only manipulated between-subjects in this dataset could be viewed as a limitation, and certainly a confirmatory replication of the modality order effect in a within-subjects design would be welcomed. However, we consider it unlikely that the modality order effect is the result of between-subject variance, as it was consistently observed in three AV and three VA experiments.

## Conclusions

In this series of six experiments, and a single trial analysis of data from five experiments, new insights to the causes of the oft-observed introspective blind spot in dual-task processing were uncovered. Interestingly, in half of the experiments, participants appeared to be aware of their dual-task processing costs. We posit that these data constitute strong evidence against the predominant model in this field, namely the conscious bottleneck model proposed by Marti et al. (2010). Indeed, these data call into question the claim that attention is required for conscious awareness (Dehaene et al., 2006). An exploratory cluster analysis illustrated that participants report a range of temporal structures when recreating the trial on a timeline in introspective PRP experiments. The LME analysis indicated that the modality of each stimulus in the trial plays an important role in introspective accuracy. We propose an alternative memory-based explanation of introspection in this attentionally demanding context, whereby introspective accuracy is determined by the memory systems involved in encoding and the time available during the trial for rehearsal of memory traces.

## Figures and Tables

**Fig. 6:**
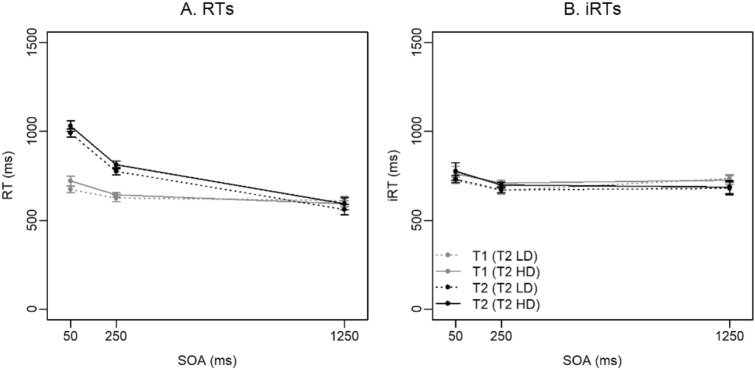
Results of Experiment 1A. Mean RT1 (grey) and RT2 (black) as a function of stimulus onset asynchrony (SOA) and Task 2 difficulty (**A**), and mean iRT1 and iRT2 as a function of SOA and Task 2 difficulty (**B**). Note: LD = low difficulty; HD = high difficulty. Error bars represent ± 1 within-subjects *SE*

**Fig. 7:**
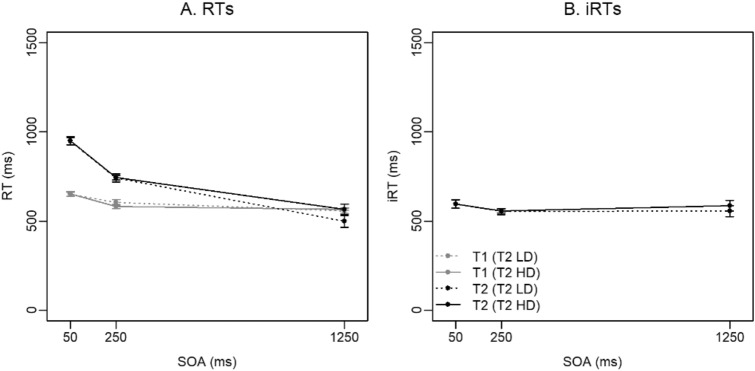
Results of Experiment 1B. Mean RT1 (grey) and RT2 (black) as a function of stimulus onset asynchrony (SOA) and Task 2 difficulty (**A**), and mean iRT2 as a function of SOA and Task 2 difficulty (**B**). Note: LD = low difficulty; HD = high difficulty. Error bars represent ± 1 within-subjects *SE*

**Fig. 8:**
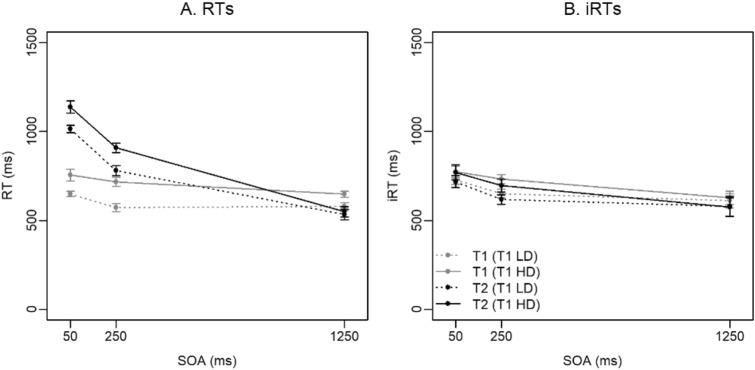
Results of Experiment 1C. Mean RT1 (grey) and RT2 (black) as a function of stimulus onset asynchrony (SOA) and Task 1 difficulty (**A**), and mean iRT1 and iRT2 as a function of SOA and Task 1 difficulty (**B**). Note: LD = low difficulty; HD=high difficulty. Error bars represent ± 1 within-subjects *SE*

**Fig. 9:**
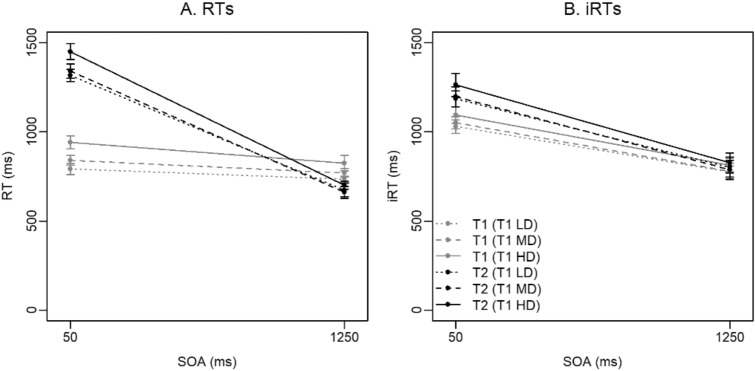
Results of Experiment 2A. Mean RT1 (grey) and RT2 (black) as a function of stimulus onset asynchrony (SOA) and Task 1 difficulty (**A**), and mean iRT1 and iRT2 as a function of SOA and Task 1 difficulty (**B**). Note: LD = low difficulty; MD = medium difficulty; HD = high difficulty. Error bars represent ± 1 within-subjects *SE*

**Fig. 10:**
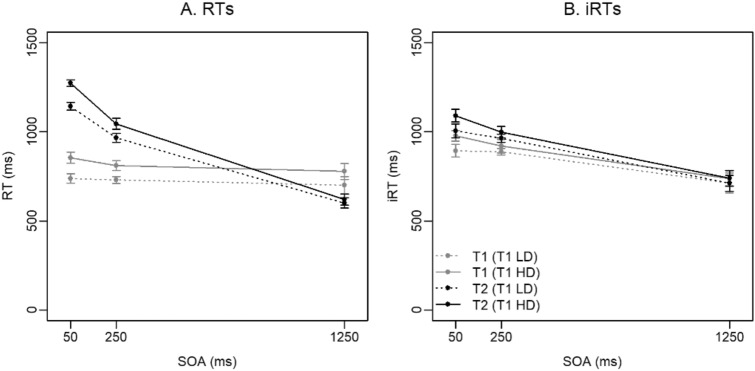
Results of Experiment 2B. Mean RT1 (grey) and RT2 (black) as a function of stimulus onset asynchrony (SOA) and Task 1 difficulty (**A**), and mean iRT1 and iRT2 as a function of SOA and Task 1 difficulty (**B**). Note: LD = low difficulty; HD = high difficulty. Error bars represent ± 1 within-subjects *SE*

**Fig. 11:**
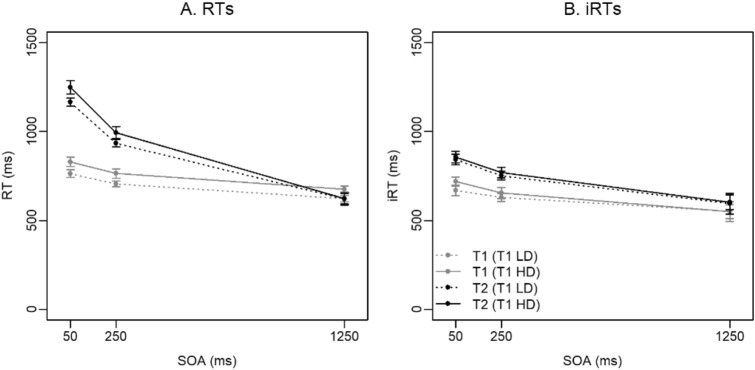
Results of Experiment 2C. Mean RT1 (grey) and RT2 (black) as a function of stimulus onset asynchrony (SOA) and Task 1 difficulty (**A**), and mean iRT1 and iRT2 as a function of SOA and Task 1 difficulty (**B**). Note: LD = low difficulty; HD = high difficulty. Error bars represent ± 1 within-subjects *SE*

**Fig. 12:**
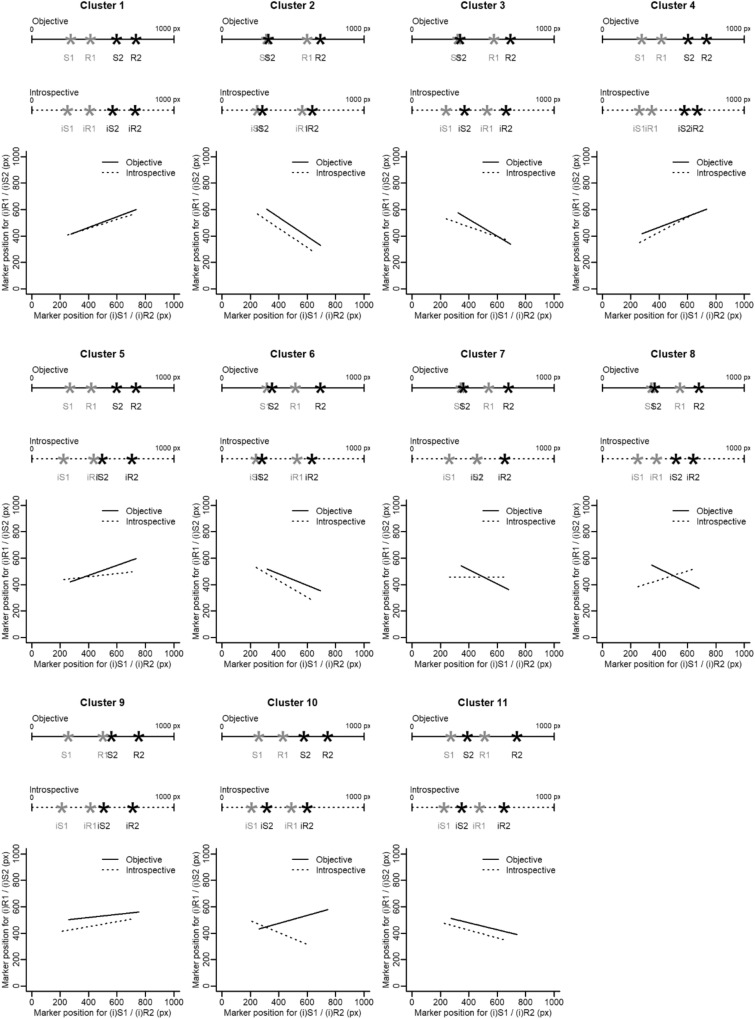
Mean objective and introspective marker positions for each cluster and the corresponding lines from which the rad_obj_ and rad_intro_ were calculated. Note: S1 = stimulus 1; S2 = stimulus 2; R1 = response to the first task; R2 = response to the second task; i = the introspective marker position

## Data Availability

The raw data and an analysis script are available at (https://doi.org/10.17605/OSF.IO/KD3R2).

## Full results from each experiment

In the main text of the manuscript we present only certain conditions of the six experiments. That is, only the shortest and longest SOA, and collapsed across difficulty manipulations. For the interested reader, we provide here the full results of each experiment. Results of the full repeated measures ANOVA for each dependent variable are presented in tabular form, and mean RTs and iRTs as a function of SOA and difficulty manipulation are presented in Figures.

### Results of Experiment 1A

Task 2 difficulty was a perceptual degradation of the visual S2. Six levels of perceptual degradation were categorised as two levels of Task 2 difficulty (low vs. high) for analysis. Table 3 and Fig. 6 show the corresponding results.

**Table 3:** Results of SOA (3) × Task 2 difficulty (2) repeated measures ANOVAs on error rates, RTs and iRTs for Experiment 1A

DV	SOA main effect	T2 Diff main effect	SOA × T2 Diff effect
T1 Error	*F*(2,30) = 0.44 *η*_*p*_^2^ = 0.03	*F*(1,15) = 0.43 *η*_*p*_^2^ = 0.03	*F*(2,30) = 0.57 *η*_*p*_^2^ = 0.04
T2 Error	*F*(2,30) = 0.50 *η*_*p*_^2^ = 0.03	*F*(1,15) = 18.85^***^ *η*_*p*_^2^ = 0.56	*F*(2,30) = 1.14 *η*_*p*_^2^ = 0.07
RT1	*F*(2,30) = 13.05^***^ *η*_*p*_^2^ = 0.47	*F*(1,15) = 1.09 *η*_*p*_^2^ = 0.07	*F*(2,30) = 1.22 *η*_*p*_^2^ = 0.08
RT2	*F*(2,30) = 69.14^***^ *η*_*p*_^2^ = 0.82	*F*(1,15) = 3.77 *η*_*p*_^2^ = 0.20	*F*(2,30) = 0.03 *η*_*p*_^2^ < 0.01
iRT1	*F*(2,30) = 1.81 *η*_*p*_^2^ = 0.11	*F*(1,15) = 0.86 *η*_*p*_^2^ = 0.05	*F*(2,30) = 1.36 *η*_*p*_^2^ = 0.08
iRT2	*F*(2,30) = 1.78 *η*_*p*_^2^ = 0.11	*F*(1,15) = 1.58 *η*_*p*_^2^ = 0.10	*F*(2,30) = 0.49 *η*_*p*_^2^ = 0.03

*Note: DV* = Dependent variable; *T1 = * Task 1; *T2 = * Task 2; *SOA =* stimulus onset asynchony

* *p* < 0.05; ** *p* < 0.01; *** *p* < 0.001

### Results of Experiment 1B

Task 2 difficulty was a perceptual degradation of the visual S2 (with two levels for analysis, as in Experiment 1A). Participants only placed the markers relating to Task 2, therefore, there are no values for iRT1. Table 4 and Fig. 7 show the corresponding results.

**Table 4:** Results of SOA (3) × Task 2 difficulty (2) repeated measures ANOVAs on error rates, RTs and iRTs for Experiment 1B

DV	SOA main effect	T2 Diff main effect	SOA × T2 Diff effect
T1 Error	*F*(2,30) = 1.40 *η*_*p*_^2^ = 0.09	*F*(1,15) = 0.48 *η*_*p*_^2^ = 0.03	*F*(2,30) = 0.35 *η*_*p*_^2^ = 0.02
T2 Error	*F*(2,30) = 1.90 *η*_*p*_^2^ = 0.11	*F*(1,15) = 38.96^***^ *η*_*p*_^2^ = 0.72	*F*(2,30) = 0.08 *η*_*p*_^2^ = 0.01
RT1	*F*(2,30) = 20.58^***^ *η*_*p*_^2^ = 0.58	*F*(1,15) = 0.20 *η*_*p*_^2^ = 0.01	*F*(2,30) = 0.90 *η*_*p*_^2^ = 0.06
RT2	*F*(2,30) = 64.59^***^ *η*_*p*_^2^ = 0.81	*F*(1,15) = 7.54^*^ *η*_*p*_^2^ = 0.33	*F*(2,30) = 4.56^*^ *η*_*p*_^2^ = 0.23
iRT1	*–*	*–*	*–*
iRT2	*F*(2,30) = 0.68 *η*_*p*_^2^ = 0.04	*F*(1,15) = 2.50 *η*_*p*_^2^ = 0.14	*F*(2,30) = 1.00 *η*_*p*_^2^ = 0.06

*Note: DV* = dependent variable; *T1* = Task 1; *T2* = Task 2; *SOA =* stimulus onset asynchony

**p* < 0.05; ***p* < 0.01; ****p* < 0.001

### Results of Experiment 1C

Task 1 difficulty was a central manipulation of the auditory S1. Tones that were furthest from the reference tone (400 and 1400 Hz) comprised the low Task 1 difficulty level and those closest to the reference tone (660 and 1029 Hz) comprised the high Task 1 difficulty level for analysis. Table 5 and Fig. 8 show the corresponding results.

**Table 5:** Results of SOA (3) × Task 1 difficulty (2) repeated measures ANOVAs on error rates, RTs and iRTs for Experiment 1C

DV	SOA main effect	T1 Diff main effect	SOA × T1 Diff effect
T1 Error	*F*(2,30) = 1.78 *η*_*p*_^2^ = 0.11	*F*(1,15) = 28.81^***^ *η*_*p*_^2^ = 0.66	*F*(2,30) = 0.50 *η*_*p*_^2^ = 0.03
T2 Error	*F*(2,30) = 7.41^**^ *η*_*p*_^2^ = 0.33	*F*(1,15) = 0.59 *η*_*p*_^2^ = 0.04	*F*(2,30) = 0.91 *η*_*p*_^2^ = 0.06
RT1	*F*(2,30) = 8.07^**^ *η*_*p*_^2^ = 0.35	*F*(1,15) = 14.85^**^ *η*_*p*_^2^ = 0.50	*F*(2,30) = 2.76 *η*_*p*_^2^ = 0.16
RT2	*F*(2,30) = 113.38^***^ *η*_*p*_^2^ = 0.88	*F*(1,15) = 12.13^**^ *η*_*p*_^2^ = 0.45	*F*(2,30) = 8.22^***^ *η*_*p*_^2^ = 0.35
iRT1	*F*(2,30) = 4.59^*^ *η*_*p*_^2^ = 0.23	*F*(1,15) = 4.24 *η*_*p*_^2^ = 0.22	*F*(2,30) = 2.99 *η*_*p*_^2^ = 0.17
iRT2	*F*(2,30) = 3.56 *η*_*p*_^2^ = 0.19	*F*(1,15) = 3.22 *η*_*p*_^2^ = 0.18	*F*(2,30) = 3.86^*^ *η*_*p*_^2^ = 0.20

*Note: DV =* dependent variable; *T1* = Task 1; *T2 =* Task 2; *SOA =* stimulus onset asynchony

**p* < 0.05; ***p* < 0.01; ****p* < 0.001

### Results of Experiment 2A

Task 1 difficulty was a perceptual manipulation of the visual S1. In this experiment only 2 SOAs were included and the difficulty manipulation examined with three levels. Table 6 and Fig. 9 show the corresponding results.

**Table 6:** Results of SOA (2) × Task 1 difficulty (3) repeated measures ANOVAs on error rates, RTs and iRTs for Experiment 2A

DV	SOA main effect	T1 Diff main effect	SOA × T1 Diff effect
T1 Error	*F*(1,15) = 0.13 *η*_*p*_^2^ = 0.01	*F*(2,30) = 56.67^***^ *η*_*p*_^2^ = 0.79	*F*(2,30) = 0.12 *η*_*p*_^2^ = 0.01
T2 Error	*F*(1,15) = 4.36 *η*_*p*_^2^ = 0.23	*F*(2,30) = 0.12 *η*_*p*_^2^ = 0.01	*F*(2,30) = 0.54 *η*_*p*_^2^ = 0.03
RT1	*F*(1,15) = 3.37 *η*_*p*_^2^ = 0.18	*F*(2,30) = 15.35^***^ *η*_*p*_^2^ = 0.51	*F*(2,30) = 1.15 *η*_*p*_^2^ = 0.07
RT2	*F*(1,15) = 161.41^***^ *η*_*p*_^2^ = 0.91	*F*(2,30) = 5.84^*^ *η*_*p*_^2^ = 0.28	*F*(2,30) = 3.98^*^ *η*_*p*_^2^ = 0.21
iRT1	*F*(1,15) = 16.30^***^ *η*_*p*_^2^ = 0.52	*F*(2,30) = 4.82^*^ *η*_*p*_^2^ = 0.24	*F*(2,30) = 0.49 *η*_*p*_^2^ = 0.03
iRT2	*F*(1,15) = 18.01^***^ *η*_*p*_^2^ = 0.55	*F*(2,30) = 5.31^*^ *η*_*p*_^2^ = 0.26	*F*(2,30) = 0.86 *η*_*p*_^2^ = 0.05

*Notes: DV=* dependent variable; *T1=* Task 1; *T2=* Task 2; *SOA=* stimulus onset asynchony

* *p* < 0.05; ***p* < 0.01; ****p* < 0.001

### Results of Experiment 2B

Task 1 difficulty was a perceptual manipulation of the visual S1 (with two levels, low and high), again with 3 SOA levels. Table 7 and Fig. 10 show the corresponding results

**Table 7:** Results of SOA (3) × Task 1 difficulty (2) repeated measures ANOVAs on error rates, RTs and iRTs for Experiment 2B

DV	SOA main effect	T1 Diff main effect	SOA × T1 Diff effect
T1 Error	*F*(2,30) = 1.69 *η*_*p*_^2^ = 0.10	*F*(1,15) = 22.93^***^ *η*_*p*_^2^ = 0.60	*F*(2,30) = 1.12 *η*_*p*_^2^ = 0.07
T2 Error	*F*(2,30) = 25.41^***^ η_p_^2^ = 0.63	*F*(1,15) = 1.96 *η*_*p*_^2^ = 0.12	*F*(2,30) = 0.89 *η*_*p*_^2^ = 0.06
RT1	*F*(2,30) = 0.59 *η*_*p*_^2^ = 0.04	*F*(1,15) = 87.39^***^ *η*_*p*_^2^ = 0.85	*F*(2,30) = 2.71 *η*_*p*_^2^ = 0.15
RT2	*F*(2,30) = 135.50^***^ *η*_*p*_^2^ = 0.90	*F*(1,15) = 40.39^***^ *η*_*p*_^2^ = 0.73	*F*(2,30) = 5.08^*^ *η*_*p*_^2^ = 0.25
iRT1	*F*(2,30) = 7.92^**^ *η*_*p*_^2^ = 0.35	*F*(1,15) = 11.61^**^ *η*_*p*_^2^ = 0.44	*F*(2,30) = 1.56 *η*_*p*_^2^ = 0.09
iRT2	*F*(2,30) = 20.19^**^ *η*_*p*_^2^ = 0.46	*F*(1,15) = 12.72^**^ *η*_*p*_^2^ = 0.46	*F*(2,30) = 1.34 *η*_*p*_^2^ = 0.08

*Note: DV =* dependent variable; *T1 =* Task 1; *T2 =* Task 2; *SOA =* stimulus onset asynchony

**p* < 0.05; ***p* < 0.01; ****p* < 0.001

### Results of Experiment 2C

Task 1 difficulty was a central manipulation of the visual S1. Numbers that were furthest from the reference number of 45 (28 and 62) comprised the low Task 1 difficulty level and those closest to the reference number (37 and 53) comprised the high Task 1 difficulty level for analysis. Table 8 and Fig. 11 show the corresponding results.

**Table 8:** Results of SOA (3) × Task 1 difficulty (2) repeated measures ANOVAs on error rates, RTs and iRTs for Experiment 2C

DV	SOA main effect	T1 Diff main effect	SOA × T1 Diff effect
T1 Error	*F*(2,30) = 0.19 *η*_*p*_^2^ = 0.01	*F*(1,15) = 1.21 *η*_*p*_^2^ = 0.07	*F*(2,30) = 0.45 *η*_*p*_^2^ = 0.03
T2 Error	*F*(2,30) = 3.15 *η*_*p*_^2^ = 0.17	*F*(1,15) = 0.33 *η*_*p*_^2^ = 0.02	*F*(2,30) = 1.48 *η*_*p*_^2^ = 0.09
RT1	*F*(2,30) = 19.24^***^ *η*_*p*_^2^ = 0.56	*F*(1,15) = 7.02^*^ *η*_*p*_^2^ = 0.32	*F*(2,30) = 0.09 *η*_*p*_^2^ = 0.01
RT2	*F*(2,30) = 107.83^***^ *η*_*p*_^2^ = 0.88	*F*(1,15) = 4.64^*^ *η*_*p*_^2^ = 0.24	*F*(2,30) = 2.71 *η*_*p*_^2^ = 0.15
iRT1	*F*(2,30) = 3.75 *η*_*p*_^2^ = 0.20	*F*(1,15) = 2.92 *η*_*p*_^2^ = 0.16	*F*(2,30) = 1.59 *η*_*p*_^2^ = 0.10
iRT2	*F*(2,30) = 10.14^**^ *η*_*p*_^2^ = 0.40	*F*(1,15) = 0.86 *η*_*p*_^2^ = 0.05	*F*(2,30) = 0.08 *η*_*p*_^2^ = 0.01

*Note: DV* = dependent variable; *T1* = Task 1; *T2 =* Task 2; *SOA =* stimulus onset asynchony

**p* < 0.05; ***p* < 0.01; ****p* < 0.001

## Cluster analysis

A cluster analysis was carried out to assess the validity of using radians to summarise the (objective or introspectively reported) temporal structure of a trial. In this analysis, single trials were used to obtain data-driven groupings of trial types based on the relationships between marker placements. To this end, trials were clustered using the k-means method (11 clusters was found to be optimal based on an iterative process). Two values per trial were entered into k-means cluster analysis: the objective radian (rad_obj_, based on the objective timing of events in the trial) and the introspective radian (rad_intro_, based on marker placements). The resulting 11 clusters were easily distinguishable and could be meaningfully interpreted. The Between Sum of Squares / Total Sum of Squares is a measure of the total variance in the dataset that is explained by the clustering solution and for this cluster analysis was 95.6%.

Descriptive statistics about each cluster are provided in Table 9 and the clusters are graphically summarised in Fig. 12, sorted by the number of trials in each cluster. Ten of eleven clusters very clearly contained a majority of either short or long SOA trials, whereas both modality orders were represented in each cluster. Negative radians indicate an event order typical of a short SOA trial (S1–S2–R1–R2), whereas positive radians indicate an event order typical of a long SOA trial (S1–R1–S2–R2).

**Table 9:** Summary of clusters, sorted by those that are composed of majority short SOA trials and long SOA trials, respectively, and number of trials in each cluster

Cluster name and description	Within cl SS^a^	% short SOA	*N* (%) AV trials	*N* (%) VA trials	Mean rad_obj_	Mean rad_intro_
Clusters with majority short SOA trials						
2: Short SOA, accurate	11	99.7	287 (7%)	1113 (16%)	− 0.61	− 0.63
3: Short SOA, inaccurate, markers evenly spaced	13	99.0	467 (12%)	866 (12%)	− 0.56	− 0.35
6: Short SOA, mostly accurate, large IRI	14	91.4	204 (5%)	711 (10%)	− 0.41	− 0.56
7: Short SOA, inaccurate, central markers grouped	18	99.0	528 (14%)	310 (4%)	− 0.48	0.004
8: Short SOA, inaccurate, markers wrong order	14	97.4	368 (9%)	259 (4%)	− 0.49	0.33
11: Mixed SOA, mostly accurate	17	62.8	125 (3%)	412 (6%)	− 0.27	− 0.29
Clusters with majority long SOA trials						
1: Long SOA, accurate	16	0.00	835 (21%)	1101 (16%)	0.38	0.33
4: Long SOA, mostly accurate, markers grouped	22	0.00	371 (10%)	802 (11%)	0.39	0.52
5: Long SOA, inaccurate, central markers grouped	16	0.00	378 (10%)	666 (9%)	0.36	0.11
9: Long SOA, accurate, central events close	18	0.87	177 (5%)	400 (6%)	0.12	0.17
10: Long SOA, inaccurate, markers wrong order	17	0.00	159 (4%)	382 (5%)	0.30	− 0.43

*Note: SOA =* stimulus onset asynchrony; *AV =* auditory S1, visual S2; *VA =* visual S1, auditory S2; *IRI* = inter-response interval

^a^Within cluster sum of squares is the squared average distance of all the points within a cluster to the cluster centroid and indicates the homogeneity of a cluster

## Footnotes

1 Another experiment using frequencies based on van Selst and Johnston (1997) was conducted first (128, 320, 2000, and 5000 Hz). The RT pattern indicated that the Task 1 difficulty manipulation did not operate as planned (i.e. there was no significant SOA × Task 1 difficulty interaction on RT2). As such, this experiment could not be considered a suitable test of the effect of delaying the end of Task 1 central processing, and alternative frequencies were selected for Experiment 1C.

2 It is pertinent to recognise here that there are multiple ways of creating these lines using alternative combinations of events as the *x*-coordinates and *y*-coordinates of the start and end of the line. Depending on which coordinate is assigned to the first and last events in the trial (typically S1 and R2), either the radian of the line or the length of the line is informative. Importantly, the correlations between measures is high, for example the rad_obj_ calculated as above correlates almost perfectly with the length of the line drawn through (S1, R2) and (S2, R1) (*r* = .97, *p* < .001).

3 Other temporal information from the trial could have been entered into the LME model to assess the influence of time on introspective accuracy, for instance the total trial duration. To assess whether this would improve the model, the same final model was built with total trial duration as a predictor instead of central gap and the AICs compared. The model with central gap had a lower AIC, indicating it is the better model.
